# RDM1 promotes critical processes in breast cancer tumorigenesis

**DOI:** 10.1111/jcmm.14425

**Published:** 2019-06-20

**Authors:** Yajun Chen, Zhengwang Sun, Tianying Zhong

**Affiliations:** ^1^ Department of Clinical Laboratory Women's Hospital of Nanjing Medical University, Nanjing Maternity and Child Health Care Hospital Nanjing China; ^2^ Department of Musculoskeletal Tumor, Shanghai Cancer Center Fudan University Shanghai China

**Keywords:** DNA damage repair, oncogenic protein, RDM1

## Abstract

Breast cancer is currently among the most common cancers in women, with almost 200,000 new cases diagnosed annually. Dysregulation of DNA repair pathways allows cells to accumulate damage and eventually mutations, with a subsequent reduction in DNA repair capacity in breast tissue, leading to tumorigenesis. One component of the DNA damage repair pathway is RAD52 motif‐containing 1 (RDM1), but the specific role of RDM1 in breast cancer and the underlying mechanism remain unclear. Here, we examined the role played by RDM1 in breast cancer cell culture using the HBL100 and MCF‐7 breast cancer cell lines. Disruption of RDM1 reduced in vitro cell proliferation and promoted apoptosis. Knockdown of RDM1 also induced up‐regulation of p53 levels, whereas RAD51 and RAD52, both involved in DNA repair, were down‐regulated. In addition, the in vivo growth of RDM1‐deficient cells was significantly repressed, suggesting that RDM1 is a novel oncogenic protein in human breast cancer cells. This study reveals a link between the DNA damage response pathway and oncogenic functionality in breast cancer. Accordingly, therapeutic targeting of RDM1 is a potential treatment strategy for breast cancer and overcoming drug resistance.

## 1 INTRODUCTION

With almost 200,000 new cases diagnosed annually, breast cancer has emerged as one of the most frequently occurring in women.^1^ Classification of breast cancers is generally based on the involvement of progesterone receptor, oestrogen receptor and human epidermal growth factor receptor two.^2^ The mean age at diagnosis for breast cancer is 61 years according to the American Cancer Society. Almost 155,000 women in the US live with metastatic breast cancer, and metastasis is present in approximately 6%‐10% of patients at diagnosis.^3^, ^4^ These tumours are complex combinations of neoplastic cells and other cell types of various origins, each occurring in a specific extracellular matrix microenvironment.^5^ Breast cancer accumulated multiple genetic abnormalities, a majority of gene therapy methods developed for the treatment of breast cancer and there approaches had less side effects compared to chemotherapy and radiotherapy.^6^ Therefore, an improved understanding of the biological mechanism of breast cancer would enable more effective and individualized therapeutic approaches.

Dysfunctional DNA damage response signalling can increase the risk of cancer. When DNA repair pathways are dysregulated, cells are predisposed to the accumulation of damage and eventually genetic mutations, subsequently reducing DNA repair capacity in the affected breast tissue and ultimately leading to tumorigenesis.^7^ One factor involved in DNA double‐strand break repair and recombination is RAD52 motif‐containing 1 (RDM1), RDM1 belongs to the gene‐binding motif containing family and its sequences show similarities to the DNA recombination and repair gene RAD52.^8^ RDM1^−/−^ cells have been reported to exhibit increased sensitivity to cisplatin.^9^ Recently, two different research groups have shown that RDM1 exhibits significant up‐regulation in human lung adenocarcinoma.^10^, ^11^ Accordingly, RDM1 and RAD52 have been proposed to share similar functions in both homologous recombination as well as DNA double‐strand break repair. Notably, RDM1 can regulate p53/RAD51/RAD52, and its down‐regulation of p53 is involved in lung adenocarcinoma.^10^ Yet, the role of RDM1 in human breast cancer remains poorly understood.

Accordingly, in this study, we knocked down RDM1 in two breast cancer cell lines and evaluated the resulting cell proliferation and apoptosis as well as other cancer‐related phenotypes. Then, using a mouse xenograft model, we further evaluated the in vivo growth of these RDM1‐knockdown cells. The resulting data support the previously identified oncogenic role of RDM1 in human lung adenocarcinoma. Our study links chemotherapeutic resistance to the involvement of RDM1 in lung adenocarcinoma oncogenesis.

## 2 MATERIALS AND METHODS

### 2.1 Cell culture

HBL100 and MCF‐7 cells were purchased from the American Type Culture Collection (Manassas, VA) and cultured in Roswell Park Memorial Institute medium supplemented with 10% foetal bovine serum and 100 U of penicillin‐streptomycin antibiotics, at 37°C under 5% CO_2_ and humidified conditions.

### 2.2 Plasmids and generation of stable knock down cells

RAD52 motif‐containing 1 was amplified from the human ES cells cDNA and cloned into PLVX‐ZsGreen vector, shRNA was bought from Sant Cruz Biotechnology.

### 2.3 RNA interference of RDM1

SiRNA was transfected using Lipofectamine 2000 reagent (Invitrogen, Calrsbad, CA) using siRNA according to the manufacturer's protocol. The siRNAs used^12^ were siRDM1‐1 (5‐UCAGAAGGCU UUGUCAGAUT T‐3) and siRDM1‐2 (5‐GCGAAUUACU ACUUUGGUUT T‐3).

### 2.4 MTT assay

2 × 10^3^ cells were seeded in 96‐well plates for the MTT assay. Cell density was measured following the instruction of Cell Viability Kit (MTT, Roche, Indianapolis, IN). The absorbance value (OD) was got in a microtitre plate reader at wavelength of 570 nm. All experiments were repeated for three times.

### 2.5 Colony formation assay

Cell proliferation was assessed using a Cell Counting Kit‐8 colorimetric assay system (Dojindo Molecular Technologies, Inc, Rockville, MD). Assays were conducted on targeted knockdown (siRDM1) cells and control cells at a density of 1 × 10^4^ cells/well. The absorbance at 450 nm was measured according to the manufacturer's instructions for each time‐point of the assay.

### 2.6 Apoptosis assay

After being seeded into six‐well plates, cells were incubated for 72 hours. Then, cells were harvested and washed in cold phosphate‐buffered saline, followed by incubation with Annexin V‐Alexa Fluor 488 conjugate and propidium iodide (100 µg/mL) for 15 minutes at room temperature. Apoptosis was then assayed using flow cytometry with excitation/emission parameters of 494/518 nm and 535/617 nm for Annexin V and propidium iodide, respectively.

### 2.7 IHC assay

For IHC, slides were boiled in Buffer TE (10 mmol/L Tris, 1 mmol/L EDTA, pH 9.0) for 20 minutes. After washing with PBS three times, the sections were permeated in H2O2 for 10 minutes, blocked with 5% BSA in PBS for 10 minutes at room temperature and incubated overnight at 4°C with RDM1 antibody. Subsequently, following three washes with PBS, slides were incubated for 1 hour with Streptavidin‐HRP peroxidase. Colour reaction product was visualized using diaminobenzidine (DAB)‐H2O2 as a substrate for peroxidase.

### 2.8 Mouse xenograft tumour model

The mouse xenograft tumour model utilized 5‐week‐old female BALB/c nude mice. Control cells and stable RDM1 knockdown (shRDM1) cells were suspended in cold phosphate‐buffered saline, and 1 × 10^6^ cells were subcutaneously injected into the right flanks of the mice. Four weeks post‐injection, mice bearing tumours were killed and the tumours were collected and measured. Tumour volume estimates were recorded based on the following formula: volume = length × width^2^ × 0.52.

### 2.9 Western blot

For Western blot analysis, equivalent amounts of protein were loaded onto sodium dodecyl sulphate‐polyacrylamide gels, and immunoblots were obtained by incubation overnight at 4°C using RDM1 (proteintech), p53(cell signalling), RAD51 (proteintech) and RAD52 (proteintech)‐specific primary antibodies. After incubation with a fluorescently labelled secondary antibody, the proteins of interest were visualized using an Odyssey Infrared Imaging System (LI‐COR, Lincoln, NE).

### 2.10 Human breast tumour expression datasets

We downloaded the raw count data of breast cancer from Xena (https://xena.ucsc.edu/). Then DESeq2 was employed to normalize and conduct differential expression analysis.^13^

### 2.11 Statistical analysis

Differences between the control and experimental samples were assessed using two‐tailed Student's *t* tests. All statistical analyses were performed using GraphPad Prism software (GraphPad Software, La Jolla, CA), with a *P* < 0.05 threshold used to assess significance. Each value is reported as the mean ± SE of the mean.

## 3 RESULTS

**Figure 1:**
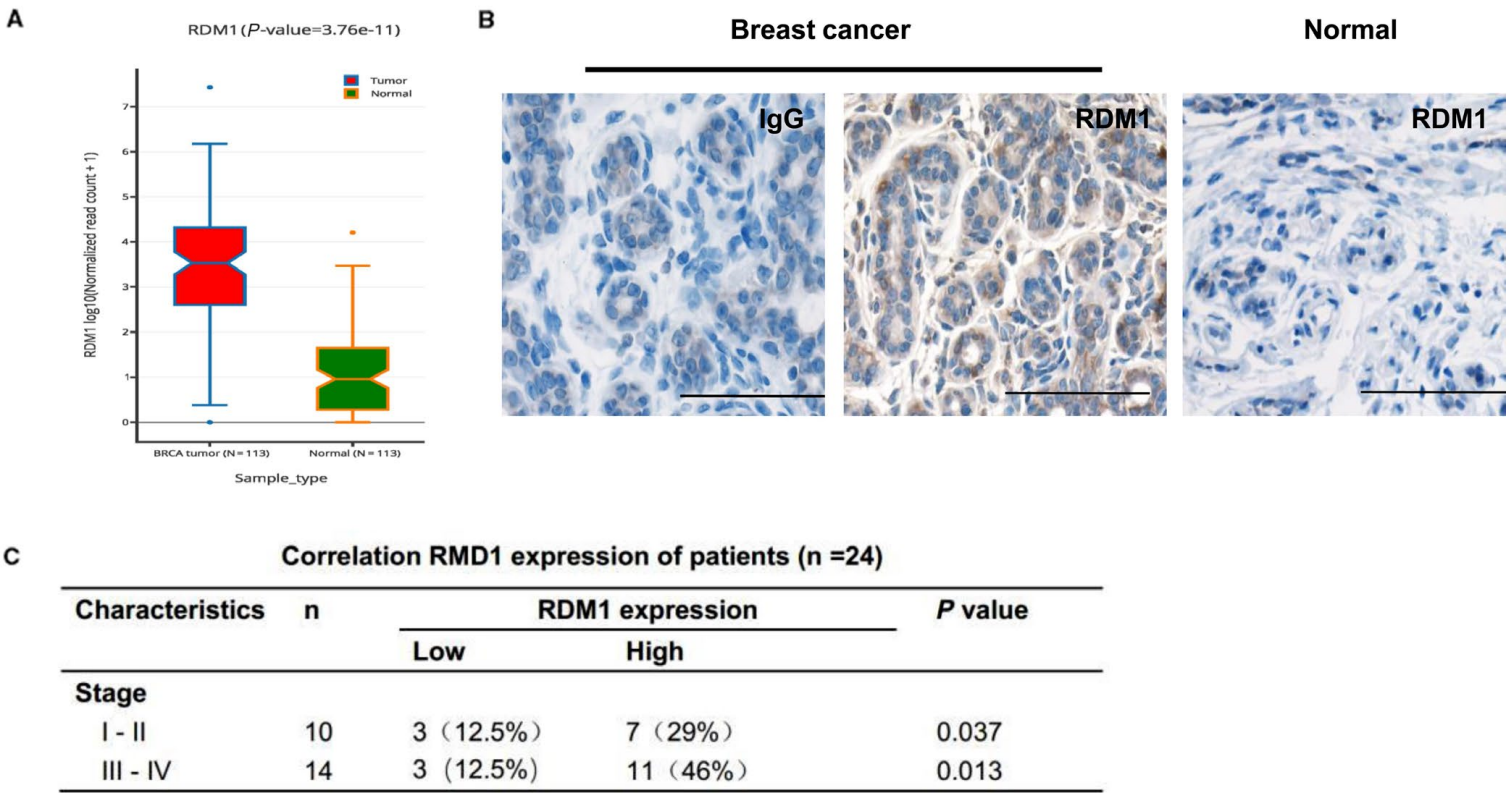
Up‐regulation of RAD52 motif‐containing 1 (RDM1) is confirmed in human breast cancer samples. (A), Multiple Oncomine analyses were performed using published datasets to examine RDM1 levels in human breast cancer. Notably, RDM1 was significantly overexpressed in breast cancer tissues compared with normal tissues (n = 113). (B,C), Immunohistochemical analysis of RDM1 expression in human breast cancer samples (original magnification, ×20) Scale bar, 50 μm. IgG was worked as a control

Multiple Oncomine analyses of RDM1 expression levels in human breast cancer based on published datasets were conducted in order to determine whether RDM1 is involved in breast cancer progression. Interestingly, RDM1 was significantly overexpressed in breast cancer tissue relative to normal tissue (Figure 1A). In accord with this bioinformatics result, the immunohistochemical analysis of breast cancer samples revealed strong positive staining for RDM1 in tumour cells but not in normal tissues (Figure 1B,C).

### 3.1 RDM1 promotes breast cancer cell growth

**Figure 2:**
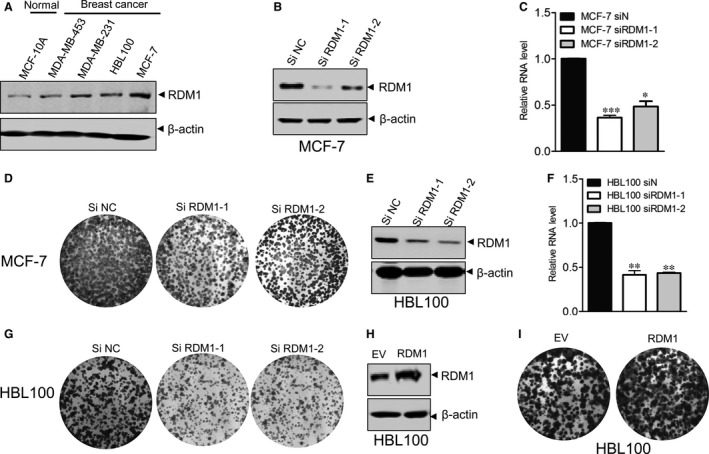
RAD52 motif‐containing 1 (RDM1) promotes breast cancer cell growth. (A), RDM1 levels in the normal breast and breast cancer cells were compared by Western blot. (B, C), Knockdown efficiency of RDM1 in MCF‐7 cells was evaluated by Western blot and real‐time PCR. (D), Clonogenic assay of MCF‐7 cells at 72 h. (E, F) Knockdown efficiency of RDM1 in HBL100 cells was evaluated by Western blot and real‐time PCR. (G), Clonogenic assay of HBL100 cells at 72 h after siRDM1 treatment. (H), RDM1 overexpression in HBL100 cells was determined by Western blotting. (I), Cell proliferation of control and RDM1 overexpression vector transfected HBL100 cells was determined by clonogenic assay. *P < 0.05; **P < 0.01; ***P < 0.001

These findings motivated a further examination of whether RDM1 was associated with breast cancer cell growth. In order to determine the function of RDM1, we detected the expression of RDM1 between breast cancer cells (MDA‐MB‐453, MDA‐MB‐231, HBL100 and MCF‐7) and normal cells (MCF‐10A). The results showed that the expression of RDM1 was obviously increased in breast cancer cells (Figure 2A). Next, RDM1 expression was determined in siNC‐treated and siRDM1‐treated MCF‐7 and HBL100 cells for 72hs. Both cell lines showed decreased protein and RNA expression of RDM1 after siRDM1 transfection (Figure 2B,C,E,F). To determine whether RDM1 promotes cell growth in breast cancer cells, we performed a colony formation assay, which revealed that colony formation was significantly reduced in siRDM1‐expressing breast cancer cell culture or overexpression condition (Figure 2D,G‐I, Figure S1A). The cell viability assay also showed that cell growth was markedly decreased in MCF‐7 and HBL100 cells after RDM1 knockdown (Figure S1B,C). Because disruption of RDM1 significantly disrupted proliferation in the breast cancer cells, we concluded that RDM1 positively regulates breast cancer cell proliferation.

### 3.2 RDM1 silencing induces cell cycle arrest and cell apoptosis

**Figure 3:**
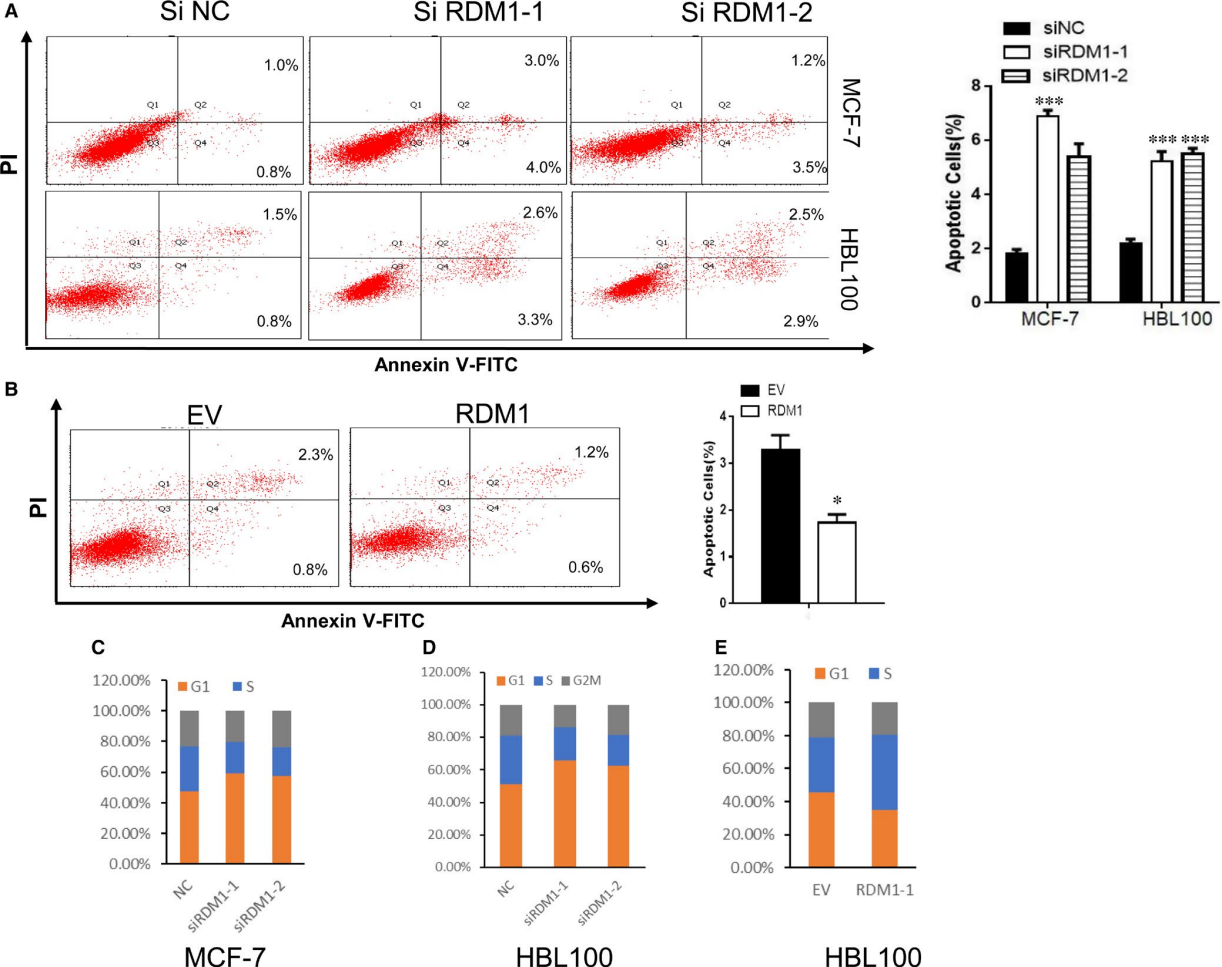
RAD52 motif‐containing 1 (RDM1) silencing induces cell apoptosis and cell cycle arrest. (A), Flow cytometry analysis of Annexin V‐FITC/propidium iodide double‐stained cell populations in order to assess apoptosis in siRDM1 MCF‐7and HBL100 cells. (B), Flow cytometry analysis of Annexin V‐FITC/propidium iodide double‐stained cell population's apoptosis in RDM1 overexpressed HBL100 cells. (C‐E), Cell cycle analysis of siRDM1 MCF‐7 (C), HBL100 (D) cells and RDM1 overexpressed HBL100 cells (E). *P < 0.05; ***P < 0.001

After confirming the role of RDM1 in breast cancer cell proliferation, we next examined the involvement of RDM1 in inducing apoptosis in breast cancer cells. Accordingly, we transiently transfected siRDM1 into MCF‐7 and HBL100 cells during a 72‐hours incubation and assessed apoptosis via double‐staining with Annexin V‐FITC and propidium iodide. The subsequent flow cytometry analysis revealed that more siRDM1 cells had undergone apoptosis relative to the control cells (Figure 3A). Conversely, overexpression of RDM1 decreased the apoptosis in HBL100 cells (Figure 3B).

DNA damage from normal metabolic processes and environmental factors induces cell cycle arrest. RDM1 is a key regulator of both DNA repair and recombination. Therefore, we analysed the cell cycle using flow cytometry, finding that RDM1‐silenced MCF‐7 and HBL100 cells exhibited significantly lower S‐phase cell populations (Figure 3C,D; Figure S2A,B). Overexpression of RDM1 up‐regulated S‐phase cell populations in HBL100 cells (Figure 3E; Figure S2C).

Taken together, RDM1 knockdown in breast cancer cells promoted cell cycle arrest as well as apoptosis, suggesting that RDM1 plays a positive role in cell viability.

### 3.3 RDM1 potentiates p53/RAD51/RAD52 signalling

**Figure 4:**
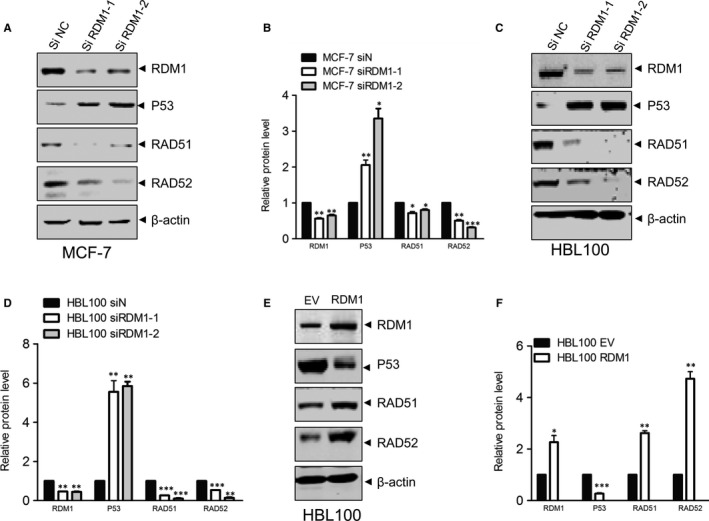
RDM1 potentiates p53/RAD52/RAD51 signalling. (A,B) Total protein was isolated from stable siRDM1 MCF‐7 cells and analysed by immunoblotting with antibodies against RDM1, p53, RAD51, and RAD52. (C,D) siRDM1 HBL100 cells were analysed by immunoblotting with antibodies against RDM1, p53, RAD51 and RAD52. (E,F) WB analysis of p53, RAD51 and RAD52 in HBL100 cells which were transfected with 4 μg PLVX‐RDM1 for 48 h compared with 4 μg EV. *P < 0.05; **P < 0.01; ***P < 0.001

RAD52 motif‐containing 1 shows similarities to RAD52. RAD52 is critical in homology‐dependent repair in yeast, but its role in mammalian cells is still unclear. Some research groups have reported that RAD52 physically interacts with RAD51 recombinase, acting as a mediator within the RAD51‐catalysed DNA strand exchange reaction.^14^ The tumour suppressor p53 is critical in stress‐induced apoptosis.^15^ It has been reported that p53 down‐regulates RAD51 expression and that the transcriptional repression of RAD51 by p53 requires specific DNA binding.^16^ Analyses of String datasets have indicated RDM1 may interact with factors that include RAD52, NIDE2 and RUNBL2.^10^ Therefore, we examined whether RDM1‐silenced cells exhibited changes in p53, RAD51 and RAD52. Notably, RDM1 knockdown readily induced the expression of p53 and decreased expression of RAD52 and RAD51 in MCF‐7 (Figure 4A,B) and HBL100 cells (Figure 4C,D). Furthermore, overexpressed RDM1 cells showed decreased p53 and increased RAD51 and RAD52 level (Figure 4E,F). Finally, we defined the potential regulation p53 by RDM1 and found p53 protein was more stable after knocking down RDM1, suggesting p53 could be regulated by RDM1 at the transcriptional level (Figure S3).

### 3.4 RDM1 knockdown inhibits breast cancer progression in a xenograft mouse model

**Figure 5:**
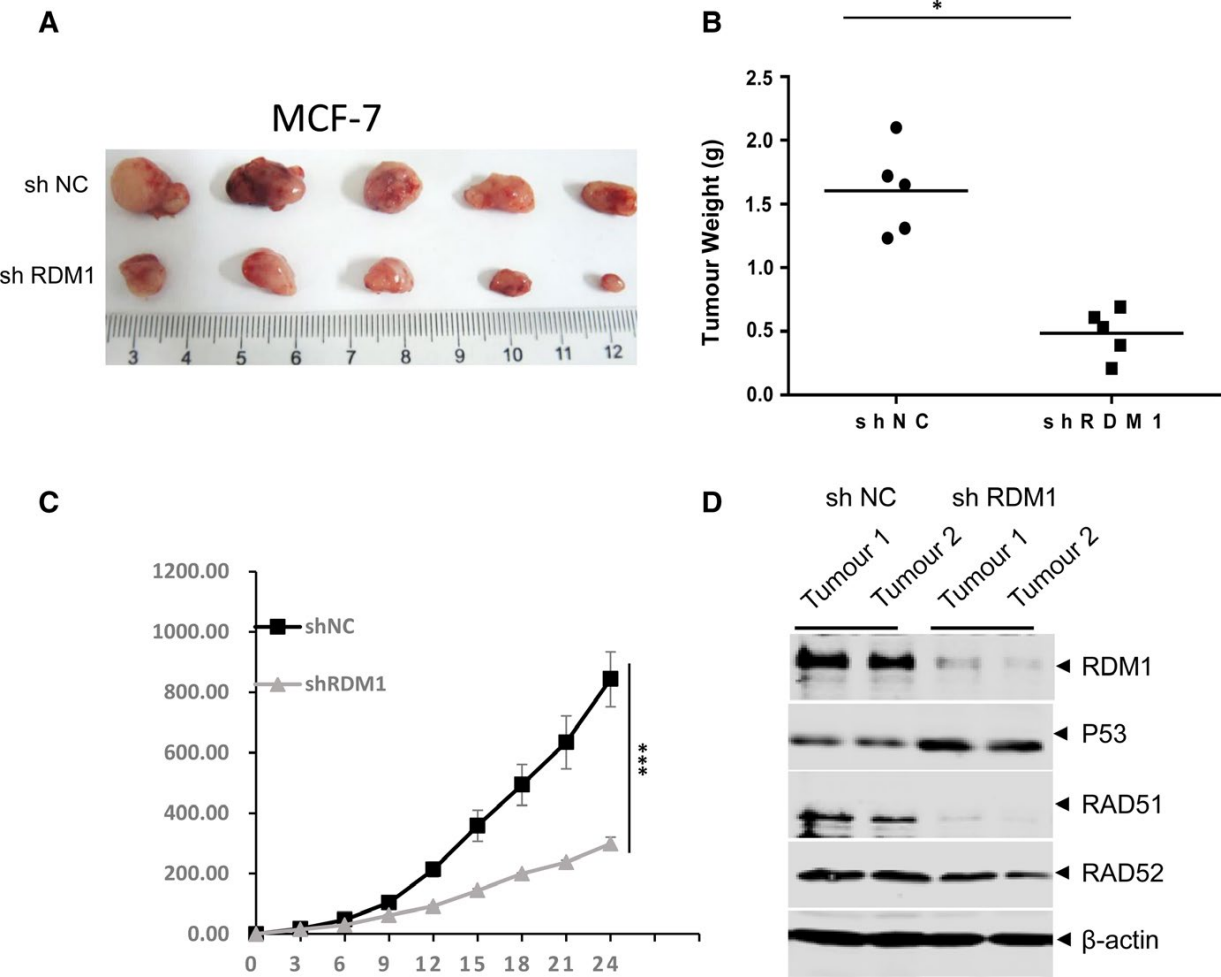
Knockdown of RAD52 motif‐containing 1 (RDM1) inhibited breast cancer progression in a xenograft mouse model. (A), A stable RDM1‐knockdown (shRDM1) MCF‐7 cell line was subcutaneously injected into 6‐week‐old immunocompromised mice. At the end of the assay, tumours were removed and photographed. (B), Tumour weights are presented as mean ± SD values (n = 5). *P < 0.05. (C), Tumour volume was determined at various time points. *P < 0.001. (D), shRDM1 MCF‐7 tumour tissues were analysed by immunoblotting with antibodies against p53, RAD51 and RAD52

To further uncover the oncogenic role of RDM1 in breast cancer cells, we injected the stable RDM1‐knockdown cell line derived from MCF‐7 cells into 6‐week‐old nude mice. Tumour sizes along the course of the in vivo growth were then recorded, revealing that RDM1 knockdown markedly decreased tumour size (Figure 5A), weight (Figure 5B) and volume (Figure 5C) relative to the control group, confirming the oncogenic role of RDM1 in breast cancer progression. In addition, the protein level of p53 was up‐regulated and the expression of RAD52 and RAD51 decreased in RDM1‐knockdown tumour tissues (Figure 5D).

## 4 DISCUSSION

Cells have various DNA damage response pathways that respond to DNA insults and thereby maintain genomic stability.^17^, ^18^ Numerous studies have indicated that the DNA damage response acts as an intrinsic barrier to the initial phases of tumorigenesis in humans^19^ and that it is frequently altered in human malignancies.^20^ In this study, we identified RDM1 as a novel oncogenic protein in breast cancer progression. The observed increase in cell apoptosis and cell cycle arrest in siRDM1 cells is a potential mechanism for the impairment of the DNA repair response. As disruption of DNA damage response signalling may impact the response to DNA‐damaging anticancer therapy, future research should determine whether DNA damage response pathways are altered in conjunction with RDM1 functionality in breast cancer cells. In addition, because the function of RDM1 is similar to that of RAD52 in DNA repair pathways in response to cisplatin‐based chemotherapy, our current findings may have a significant impact on the identification of critical factors that can be targeted to mitigate chemotherapeutic resistance to cisplatin‐based treatment.

The potent tumour suppressive activity of p53 occurs through the induction of apoptosis, senescence, cell cycle arrest and metabolism regulation. In addition, recent work suggests that p53 binds to the nucleotide excision repair factors XPD and XBD, acting to modulate their DNA repair activity.^21^ Additionally, p53 can direct apoptosis of a damaged cell when these cell cycle arrest and DNA repair functions fail to restore a genome to its wild‐type state.^22^ Notably, our study suggests that RDM1 regulates the expression of p53, as RDM1‐knockdown cells showed significant p53 up‐regulation. While the in vivo interaction between hRad51 and p53 has already been described,^23^ in order to infer the functional consequences of this binding, it may be useful to define the binding domains of both hRad51 and p53.^24^ RDM1 and RAD52 have been proposed to share similar functions in homologous recombination and DNA double‐strand break repair. We also determined that RDMI knockdown decreased RAD52 and RAD51 expression, suggesting a relationship between RDM1 and p53/RAD52/RAD51 signalling. Finally, because our initial Oncomine analyses demonstrated that RDM1 is highly expressed in breast cancer samples from patients, we propose that RDM1 may be a potential prognostic breast cancer marker. Further research should be conducted to conclusively assess the expression of RDM1 in human tissues and its relationship with clinical prognoses.

In conclusion, this study offers important insights into the mechanism by which RDM1 is involved in breast cancer. The observed function of RDM1 in two different cell lines indicates that it is an important factor in cell apoptosis, cell growth and the cell cycle. More importantly, RDM1 was determined to potentially regulate p53/RAD52/RAD51 signalling. Therefore, we predict that therapeutic targeting of RDM1 could be a promising strategy for clinical breast cancer therapy and overcoming drug resistance.

## CONFLICT OF INTEREST

The authors declare that they have no competing interests.

## ETHICS APPROVAL AND CONSENT TO PARTICIPATE

All procedures involving animals were approved by the Animal Ethics Committee of the Women's Hospital of Nanjing Medical University (Approval no. 2019‐C004).
