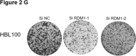# Correction to RDM1 promotes critical processes in breast cancer tumorigenesis

**DOI:** 10.1111/jcmm.17752

**Published:** 2023-07-04

In Y Chen et al^1^, the published article contains errors in Figure 2G. The corrected Figure 2G is below. The authors confirmed that the conclusion of this article remain unchanged.