# Supplementary figures and images for: RDM1 promotes critical processes in breast cancer tumorigenesis
Source: J Cell Mol Med. 2019 Jun 20;23(8):5432–9. doi: 10.1111/jcmm.14425 (PMC6653204; doi:10.1111/jcmm.14425)

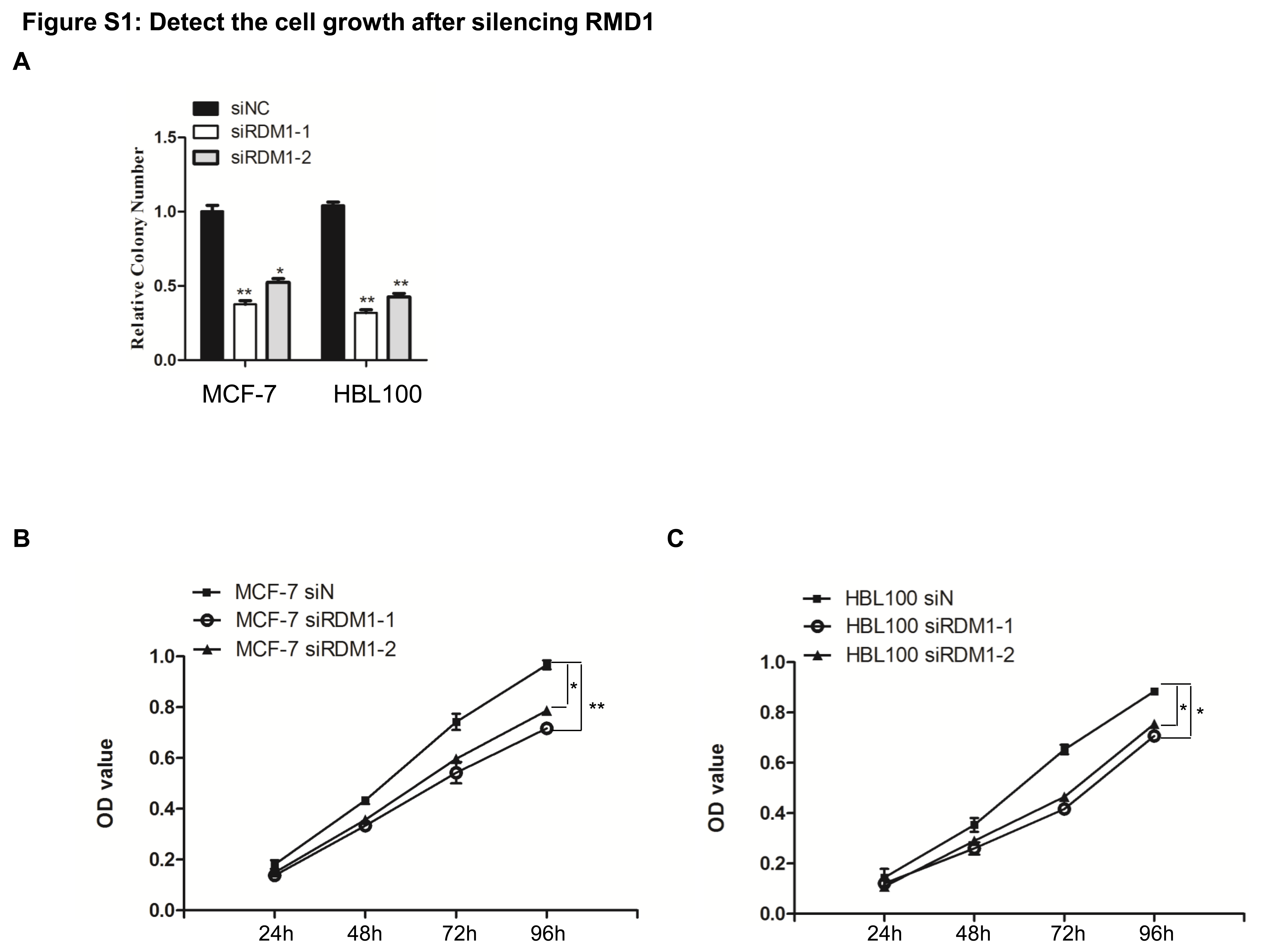

Supplement: Supplementary file 1 [file JCMM-23-5432-s001.tif]

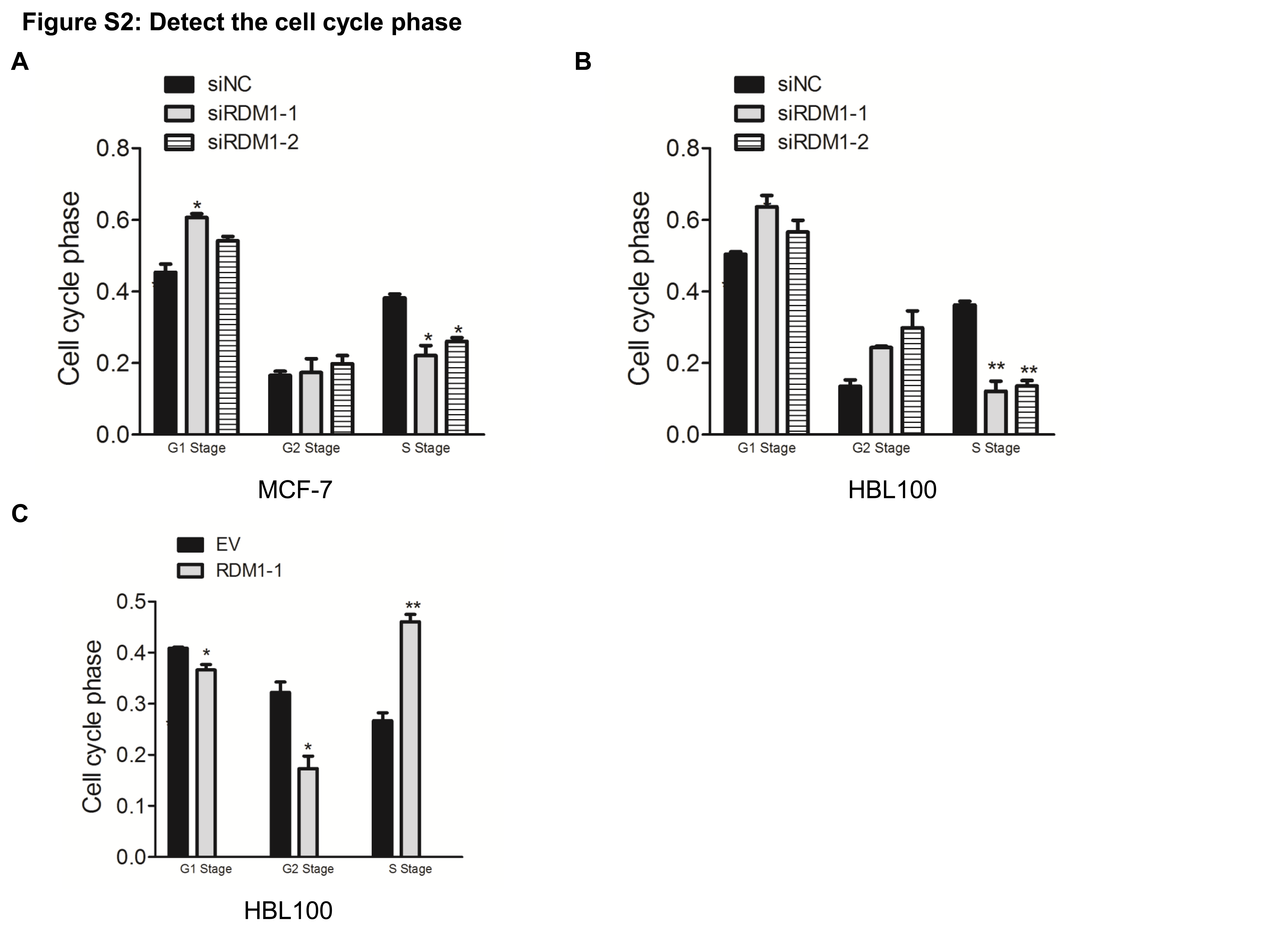

Supplement: Supplementary file 2 [file JCMM-23-5432-s002.tif]

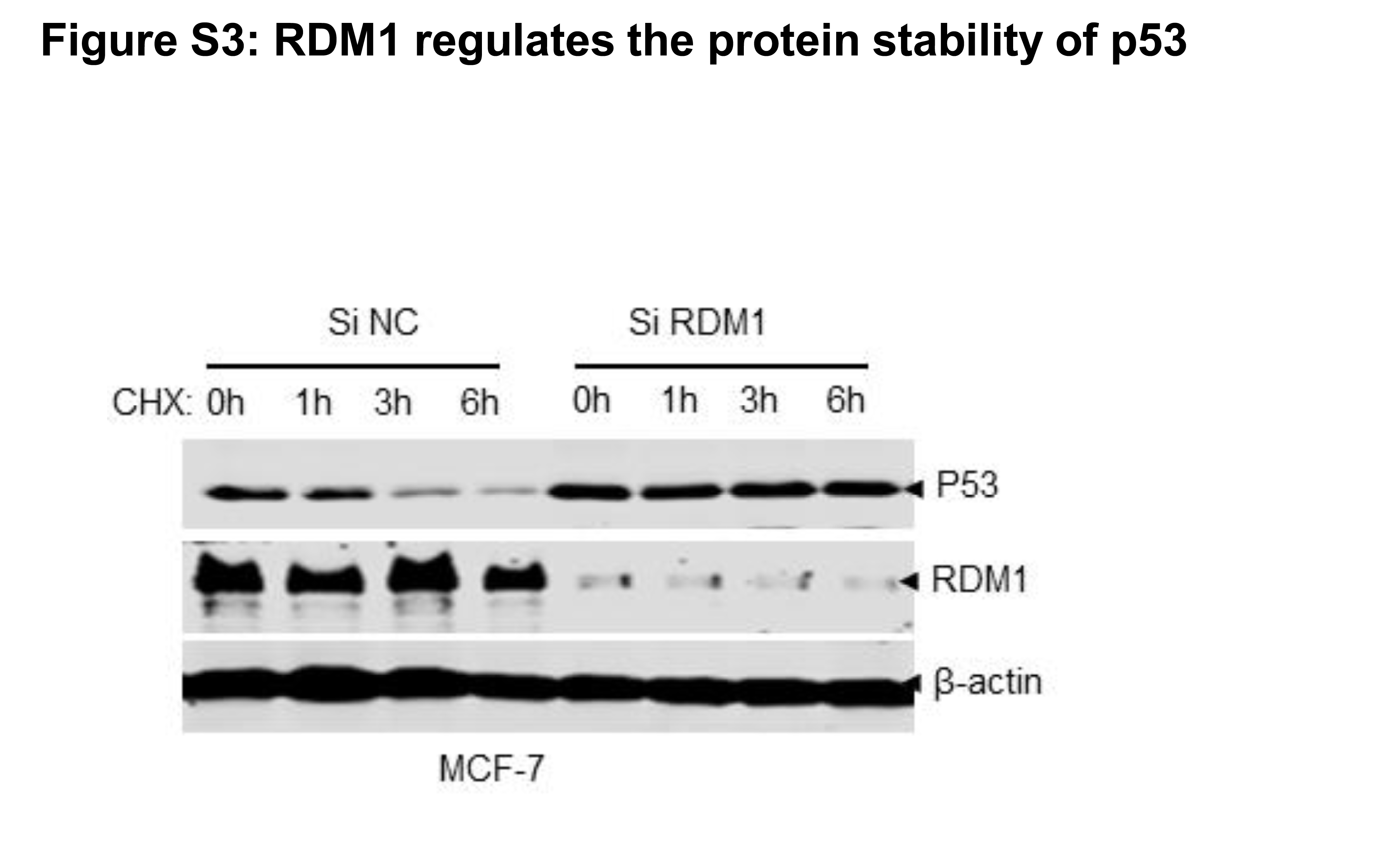

Supplement: Supplementary file 3 [file JCMM-23-5432-s003.tif]
