# Supplementary material for: RDM1 promotes critical processes in breast cancer tumorigenesis
Source: J Cell Mol Med. 2019 Jun 20;23(8):5432–9. doi: 10.1111/jcmm.14425 (PMC6653204; doi:10.1111/jcmm.14425)
Supplement: Supplementary file 4 [file JCMM-23-5432-s004.docx]

**Figure S1: Detect the cell growth after silencing RMD1**

(A) Quantitive analysis of clonogenic assay in MCF-7 and HBL100 cells at 72 h after siRDM1 treatment.

(B) After 72 hours post transfection with siRNA against RDM1 or siNC, MCF-7 and HBL100 cell viability was monitored by MTT assay at the indicated times.

**Figure S2: Detect the cell cycle phase**

(A-B) Quantitive analysis of cell cycle in MCF-7 (A) and HBL100 (B) cells at 72 h after siRDM1 treatment. * p<0.05; **, p<0.01.

(C) Quantitive analysis of cell cycle in RDM1 overexpressed HBL100 cells. * p<0.05; **, p<0.01.

**Figure S3: RDM1 regulates the protein stability of p53**

Western blots analysis of P53 in MCF-7 cells after knockdown of RDM1 with Cycloheximide (CHX) treatment.
